# Ecological validity of walking capacity tests following rehabilitation in people with multiple sclerosis

**DOI:** 10.1371/journal.pone.0220613

**Published:** 2019-08-01

**Authors:** Rainer Ehling, Gabriel Bsteh, Andreas Muehlbacher, Kay Hermann, Christian Brenneis

**Affiliations:** 1 Department of Neurology, Clinic for Rehabilitation Münster, Münster, Austria; 2 Karl Landsteiner Institut für Interdisziplinäre Forschung am Reha Zentrum Münster, Münster, Austria; 3 Clinical Department of Neurology, Medical University of Innsbruck, Innsbruck, Austria; Emory University, UNITED STATES

## Abstract

**Background:**

Walking capacity tests are commonly used to evaluate interventions aiming at reducing walking impairment in people with multiple sclerosis (pwMS). However, their ecological validity has recently been questioned. The aim of the present study was to investigate the ecological validity of the 2- and 6-minutes walking tests (2MWT and 6MWT) and the timed 25-foot walk (T25FW) after 28 days of multidisciplinary inpatient rehabilitation (MIR) in pwMS using accelerometry.

**Methods:**

PwMS wore an accelerometer on 7 consecutive days within a 14-day period prior to MIR, performed 2/6MWT and T25FW at the beginning and at the end of MIR, followed by another 7 consecutive days of accelerometry.

**Results:**

Significant improvements in 2/6MWT and T25FW after MIR in a cohort of 76 pwMS (mean age = 47.9, SD 8.3 years) were overall correlated to a significant gain in everyday life mobility (total steps/day). However, the correlation was strongly dependent on pre-existing walking disability defined by EDSS and only pwMS with “mild” walking impairment (EDSS 2–3.5) were able to transfer benefits measurable by walking capacity tests into improved everyday life mobility, while pwMS with “moderate to severe” walking disability (EDSS 4–6.5) were not.

**Conclusion:**

Ecological validity of changes in walking capacity tests following MIR is strongly dependent on pre-existing walking impairment.

## Introduction

Walking impairment in people with multiple sclerosis (pwMS) is highly prevalent, occurs early in the disease course, and has a profound negative effect on health-related quality of life (HRQoL) [1,2]. Furthermore, it was associated with an increased risk for cardiovascular diseases [3]. Therefore, treatment of walking limitations in pwMS is crucial and addressed by pharmacotherapy and rehabilitation interventions. It is therefore of importance to be able to measure walking disability in pwMS with confidence.

The Expanded Disability Status Scale (EDSS) is an established clinical measure of disability in MS; it assesses disability using maximal walking distance and the need for a walking aid in the range between 4.0 and 7.0 [4]. However, substantial intra- and inter-rater variability, low sensitivity to change and the non-linear measurement limit the use of the EDSS for the assessment of walking disability [5–7]. Short and long walking tests, i.e. the timed 25-foot walk (T25FW), the 2-minute walking test (2MWT) and the 6-minute walking test (6MWT), but also the self-reported Multiple Sclerosis Walking Scale-12 (MSWS-12) have proven as objective but also valid and reliable walking-specific measures [8,9]. Changes in the T25FW that occur over time [10,11] or after medical treatment [12,13] that exceed ≥20% are considered as being clinically meaningful, irrespective of a potential statistically significant difference. In addition, reference values for clinically meaningful changes occurring over time or after rehabilitation interventions have also been reported for long walking tests and the MSWS-12 [14,15]. However the ecological validity of these walking-specific measurements, that is the extent to which changes in these tests can be generalized to everyday life, has recently been questioned. Concerns arise from the observation that uninterrupted walking over 2 or 6 minutes is very uncommon in daily lives of pwMS thereby challenging its real life relevance [16,17]. In addition, short and long walking tests mainly sample walking capacity in an artificial setting instead of real-life walking performance of pwMS, which is further reflected by the T25FW, 2MWT and 6MWT correlating only weakly with everyday life walking reported in cross-sectional studies [18,19]. Though only improvement in walking tests that also depict a change in everyday life walking can slow worsening of symptoms, reduce the risk for cardiovascular diseases and enhance participation in pwMS [20,21].

Free-living accelerometry offers a valuable opportunity for gaining objective, reliable and ecologically valid mobility data of pwMS by detecting raw acceleration and activity counts based on the intensity of body displacement [22–26]. In comparison to pedometers, accelerometers present higher accuracy across different walking speeds in pwMS, independent of mobility impairment [27], although algorithms used for estimating walking speed from raw accelerometer data are crucial [28].

The aim of the present study was to longitudinally investigate whether an improvement in short and long clinical gait tests after multidisciplinary inpatient rehabilitation (MIR), the current golden standard in treating mobility impairment, is related to an increased everyday life mobility of pwMS according to the degree of walking disability.

## Methods

### Study design and participant recruitment

The study was approved by the ethics committee of the Medical University Innsbruck, Austria, and all participants provided written informed consent (AN5228 329/4.11).

Study participants were consecutively recruited from all pwMS referred to MIR to the Clinic for Neurological Rehabilitation Münster, Austria, which is the primary rehabilitation clinic in the region of Tyrol providing MIR for patients with neurological diseases and therefore likely to include most pwMS from this geographic area.

Applied eligibility criteria were age (18–70 years), diagnosis of MS of any disease subtype [29], a score on the expanded disability status scale (EDSS) of ≥2 and ≤6.5 and a primary rehabilitation goal of improving mobility as defined by the international classification of functioning, disability and health (ICF) [30]. To ensure a clinically stable MS cohort and to rule out a potential prolonged effect of methylprednisolone on motor recovery, pwMS, who had a relapse 30 days prior to study entry, were excluded in line with similar studies [11,15]. Further exclusion criteria concomitant diseases associated with mobility impairment or severe cognitive impairment (mini mental status examination<27). To account for the impact of walking disability on mobility outcome measures, pwMS were grouped into “mild” (EDSS<4) and “moderate to severe” (EDSS≥4) as suggested previously [15].

### Measurements

**Fig 1:**
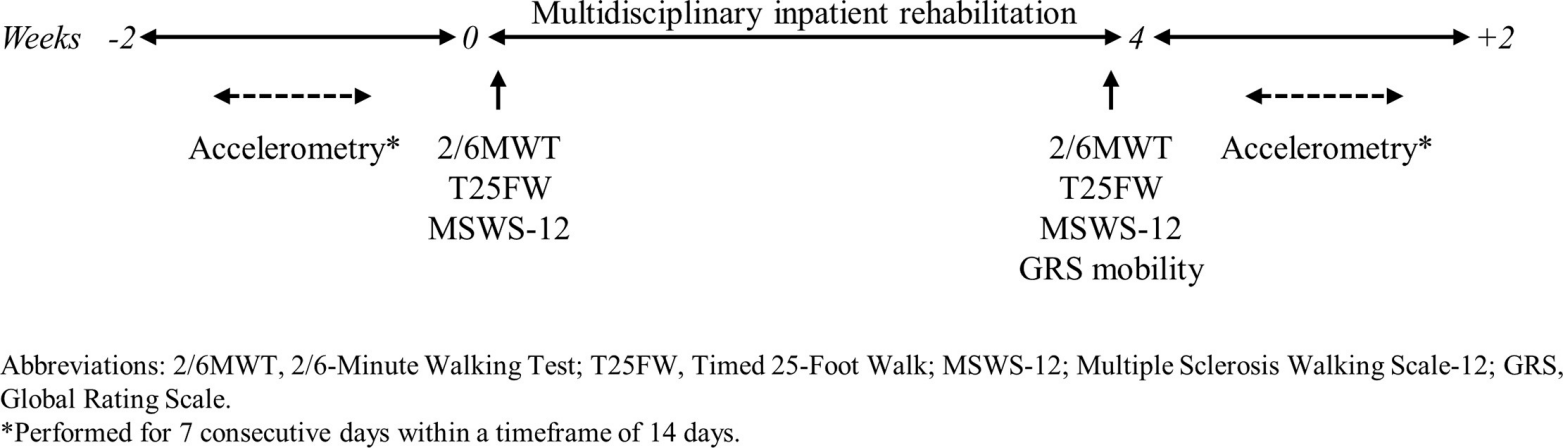
Time schedule of study procedures.

Measurements were performed according to the time schedule outlined in Fig 1. Compliance with standardized protocols, including detailed test procedures, verbal instructions and level of encouragement during testing was ensured by two experienced raters (AM, KH), who have participated in previous multicenter studies applying walking tests.

#### 2-minute and 6-minute walking test (2MWT and 6MWT)

The 6MWT was chosen as a sub-maximal aerobic walking exercise test. To account for the restricted walking performance of participants with EDSS values ≥4, the 2MWT was chosen, since it has recently been suggested as an appropriate replacement because of its strong correlation with the 6MWT [18,31]. Participants were asked to walk as fast and as far as possible back and forth along a 30-m hallway, turning around cones at each end, using their usual walking aids and/or orthotics [32]. The distance was recorded to the nearest meter, with longer distances indicating better mobility.

#### Timed 25-Foot Walk (T25FW)

After the 2MWT or 6MWT, participants performed two T25FWs less than five minutes apart as detailed in the MSFC recommendations [33]. Participants were instructed to walk the T25FW safely, but at fastest possible speed. Walking times were averaged and transformed into speeds (m/s).

#### Accelerometry

Everyday walking behavior was measured using ActiGraph accelerometers (model GT3X) according to the manufacturers’ protocol (ActiGraph, Pensacola, Florida). ActiGraph GT3X uses microelectromechanical sensing resulting in an electric signal proportionate to the force acting upon it during movement. Acceleration is captured by a 12 bit analog to digital converter at a rate of 30 Hz and merged over one-minute sampling intervals. Post-processing in ActiLife6 software results in step counts that are based on accelerometer data collected on the vertical axis only (ActiGraph, Pensacola, Florida). For a reliable estimate of everyday walking of pwMS only data of at least three or more available days with a minimum daily wear time of 600 minutes was included. The decision for three or more days was based on a previously published reliability analysis supporting this threshold consistently yielding an intra-class correlation coefficient exceeding 0.80 [34].

#### Self-reported mobility and fatigue questionnaires

The 12 Item MS Walking Scale (MSWS-12) measures walking ability by assessing the impact of MS on 12 aspects of walking function and quality (walking, running, climbing stairs, standing, balance, distance, effort, support needed indoors and outdoors, speed, smoothness, and concentration needed to walk) that have been identified as being important by pwMS [35]. To ease interpretation, scores of all 12 items were summed (ranging from 12–60) and transformed (transformed score = [(observed score—12)/60–12)] * 100) to 0–100 (minimum to maximum walking disability). A negative MSWS-12 change score implies that the respondent perceived walking ability to have improved.

A seven-point Likert-type global rating of change scale (GRS) on mobility from patient perspective was further applied after completing MIR. The GRS question asked, “Compared to before rehabilitation, how would you rate your mobility now?” Possible response categories were: very much improved, much improved, improved, no change, worse, much worse, very much worse.

The impact of MS-related fatigue on mobility was evaluated using the Fatigue Severity Scale prior and after MIR [36].

### Multidisciplinary inpatient rehabilitation

Following the standard procedure at the facility, MIR at the Department of Neurology, Clinic for Rehabilitation Muenster, Muenster, Austria was multidisciplinary and organized as four weeks of continuous hospitalization with 25 days of scheduled rehabilitation. According to the primary rehabilitation goal of improving mobility, the program consisted predominantly of individual and group physiotherapy. Group physiotherapy was supervised and guided by a physiotherapist, included a maximum number of ten participants and consisted of balance and gait training, endurance and resistance training. Adherence to the program was ensured by the therapist. MIR was complemented with occupational and speech therapy, sessions with neuropsychologists, consultations with the neurologist and educational lessons on different topics (e.g. pharmacological and non-pharmacological treatment strategies in MS).

### Statistical analysis

Variables are presented as absolute numbers and percentages, mean and standard deviation (SD) or median and inter-quartile range (IQR). Participants were categorized according to walking distance restriction as either “mild” (EDSS<4) or “moderate to severe” (EDSS≥4). Differences between groups in categorical variables were evaluated using chi-square test. Numeric variables were analyzed by independent t-test or Mann-Whitney U test depending on normal distribution tested by Kolmogorov-Smirnov-test. One-way analysis of variance for repeated measures and Wilcoxon matched-pairs test were used to examine the significance of changes in walking measures after MIR. The Pearson test was used for correlation analysis of EDSS and change in steps/day and steps performed in one-minute intervals. The influence of mobility outcome measures on the change in steps/day after MIR was evaluated by multivariate linear regression models correcting for age, gender, disease duration and EDSS.

A sample size calculation was not undertaken as the study was exploratory in intent and there is no definitive guidance on calculating sample size for ecological validity studies. Significance was based on a p-value of <0.05 and data was analyzed using SPSS Statistics-Version 22.0 (SPSS Inc., Chicago, IL, USA).

## Results

### Participants and drop outs

Of the 86 consecutively recruited pwMS, ten had to be excluded from the final analysis: three pwMS could not be assessed after MIR because of unforeseen medical circumstances leading to premature termination of MIR (clinical relapse, fracture, pneumonia); accelerometer data from another six pwMS was either insufficient (less than 3 days with a wear time of 600 minutes/d; n = 5) or worn after a period of more than 14 days after MIR (n = 1); one patient refused to wear the accelerometer after MIR. There were no significant differences between the ten excluded pwMS and the 76 pwMS finally analyzed in the study with respect to age, gender, disease duration, disease subtype and EDSS (S1 Table).

A median EDSS of 3.0 indicated moderate disability in the final cohort. There was a pronounced female predominance (65%). The majority of pwMS was diagnosed with relapsing-remitting MS (61%) and on a disease modifying treatment (DMT; 52%). Table 1 details baseline characteristics of the whole cohort.

**Table 1:** Baseline characteristics of study cohort.

		Whole Group	"Mild" (EDSS 2–3.5)	"Moderate-severe" (EDSS 4–6.5)	*p*-value
N		76	49	27	
Age (years)^1^		47.9 (8.3)	47.4 (8.7)	48.8 (7.7)	0.483*
Females^2^		49 (64.5)	32 (65.3)	17 (63.0)	0.838**
Body mass index (kg/m2)^1^		25.1 (4.2)	25.4 (3.9)	24.7 (4.8)	0.518*
Status of employment^2^					
	unemployed	43 (56.6)	26 (53.1)	17 (63.0)	0.689**
	partial employment	9 (11.8)	6 (12.2)	3 (11.1)	
	full employment	24 (31.6)	17 (34.7)	7 (25.9)	
Disease duration (years)^1^		11.9 (8.8)	11.3 (9.0)	12.9 (8.6)	0.337***
Disease course^2^					
	Relapsing remitting	46 (60.5)	39 (79.6)	7 (25.9)	<0.001**
	Secondary progressive	13 (17.1)	1 (2.0)	12 (70.4)	
	Primary progressive	17 (22.4)	9 (18.4)	8 (29.6)	
EDSS^3^		3.0 (2.0–5.5)	2.0 (2.0–3.0)	6.0 (5.5–6.5)	<0.001***
Disease modifying treatment^2^					
	Treated	40 (52.6)	27 (55.1)	13 (48.1)	<0.561**
	Untreated	36 (47.4)	22 (44.9)	14 (51.9)	** **
T25FW (meters/second)^1^		1.5 (0.6)	1.8 (0.4)	0.9 (0.4)	<0.001*
Daily step count^1^		5258.2 (3148.1)	6529.9 (3046.4)	2950.4 (1696.0)	<0.001***
MSWS-12^1^		43.9 (29.6)	34.9 (24.3)	65.0 (30.7)	0.001*
FSS^1^		4.9 (1.6)	4.7 (1.5)	5.2 (1.7)	0.232*
Accelerometer wear time (min)^1^		822.7 (92.9)	824.6 (93.4)	819.3 (93.5)	0.987***

Abbreviations: EDSS, Expanded disability status scale; T25FW, Timed 25-Foot Walk; MSWS-12; Multiple Sclerosis Walking Scale-12; Fatigue Severity Scale.

Values are given as

^1^mean and standard deviation

^2^absolute number and percentage

^3^median and interquartile range.

Differences between walking disability subgroups were evaluated using

*independent t-test

**chi-square test or

***Mann-Whitney U test depending on normal distribution.

### Content of multidisciplinary inpatient rehabilitation

PwMS received a mean of 712 minutes (SD 222) of individual and 846 minutes (SD 222) of group-based physiotherapy; and 410 minutes (SD 252) of individual therapy other than physiotherapy and 311 minutes (SD 204) of group-based therapy other than physiotherapy. Content of MIR did not significantly differ between disability subgroups (S2 Table).

### Baseline comparison between disability subgroups

At baseline, subgroups differed significantly with respect to T25FW (*p* <0.001), MSWS-12 (*p* = 0.001) and total steps count per day (*p* <0.001). Further, a significant larger proportion of people with primary and secondary progressive MS was found in the “moderate to severe” walking disability subgroup (*p*<0.001). These significant disparities among the subgroups indicate an EDSS cut-off score of 4.0 to be justified for differentiating pwMS with “mild” versus “moderate to severe” walking disability. Subgroups did not differ significantly with respect to age, gender, BMI, status of employment, disease duration, presence of DMT, fatigue and adherence to seven-days accelerometry (Table 1).

### Correlation of everyday walking behavior with short and long walking tests and self-reported questionnaires

Total steps count per day of the investigated MS-cohort was positively correlated with 2MWT (correlation coefficient *ρ* = 0.47; p = 0.018), 6MWT (*ρ* = 0.49; p<0.001), T25FW (*ρ =* 0.63; p<0.001) and negatively correlated with EDSS (*ρ* = -0.73; p<0.001) and MSWS12 (*ρ* = -0.49; p<0.001).

### Effect of multidisciplinary inpatient rehabilitation on mobility outcome measures

Objective clinical gait testing, i.e. T25FW showed statistically significant improvement in the whole cohort (p<0.001), indicating overall better performance after MIR. In addition, total steps count per day showed statistically significant improvement after MIR (p = 0.033; Table 2). Improvement of total steps count per day was negatively correlated with EDSS (*ρ* = -0.298; p<0.001).

**Table 2:** Changes in walking measures and self-reported questionnaires after multidisciplinary inpatient rehabilitation.

		Pre-MIR	Post-MIR	Change^a^	*p-value*
Whole Group (n = 76)					
	T25FW (meters/second)	1.5 (0.6)	1.6 (0.7)	0.2 (0.3)	<0.001*
	Daily step count	5258.2 (3148.1)	5582.0 (3172.9)	323.8 (1331.3)	0.033*
	MSWS-12	43.9 (29.6)	34.4 (24.5)	-9.5 (20.6)	0.002**
	FSS	4.9 (1.6)	4.2 (1.7)	-0.7 (1.3)	<0.001**
"Mild" walking disability (EDSS <4; n = 49)					
	T25FW (meters/second)	1.8 (0.4)	1.9 (0.5)	0.2 (0.3)	0.001*
	6MWT (meters)	489.9 (91.7)	538.8 (96.6)	48.9 (48.7)	<0.001**
	Daily step count	6529.9 (3046.4)	7069.4 (2765.7)	539.5 (1530.1)	0.017*
	MSWS-12	34.9 (24.3)	25.5 (19.5)	-9.5 (21.7)	0.014**
	FSS	4.7 (1.5)	3.8 (1.6)	-0.8 (1.4)	0.001**
"Moderate-severe" walking disability (EDSS 4–6.5; n = 27)					
	T25FW (meters/second)	0.9 (0.4)	1.0 (0.4)	0.1 (0.2)	0.015*
	2MWT (meters)	85.6 (34.5)	100.4 (46.8)	14.8 (35.1)	0.008**
	Daily steps count	2950.4 (1696.0)	2882.8 (1784.1)	-67.6 (737.2)	0.442*
	MSWS-12	65.0 (30.7)	55.1 (22.7)	-9.7 (18.5)	0.049**
	FSS	5.2 (1.7)	4.8 (1.9)	-0.5 (1.0)	0.032**

Abbreviations: T25FW, Timed 25-Foot Walk; 6/2MWT, 6/2-Minute Walking Test; MSWS-12; Multiple Sclerosis Walking Scale-12; Fatigue Severity Scale.

Values are given as mean and standard deviation.

Analyzed using *Wilcoxon’s matched-pairs test and **one-way analysis of variance for repeated measures.

^a^ Improvement is indicated by positive change scores except for MSWS-12 and FSS

When grouping pwMS according to walking disability, the subgroup with “mild” impairment (EDSS<4) improved in 6MWT and T25FW (p<0.001 and p = 0.001 respectively) but also in the total number of steps performed per day (p = 0.017). However, while the subgroup with “moderate to severe” walking disability (EDSS≥4) also improved in the 2MWT and in the T25FW (p = 0.008 and p = 0.015 respectively), accelerometer data was comparable to pre-MIR values and did not differ significantly (Table 2).

### Low EDSS is an independent predictor for an increase in everyday mobility after MIR

In a multivariate model, lower EDSS was found to be an independent predictor of a greater gain in total steps count per day after MIR, while age, gender and disease duration were not (Table 3).

**Table 3:** Multivariate model of change in total steps count after multidisciplinary inpatient rehabilitation.

	Coefficient^1^	95% confidence interval	*p*-Value
Age	0.034	-1.703–2.285	0.772
Female	-0.074	-2.648–1.340	0.515
EDSS	-0.272	-4.379 - -0.392	0.020
Disease duration	-0.133	-3-131–0.857	0.259

^1^Calculated by multivariate linear regression model indicating an association between lower EDSS scores and a higher increase in total steps count after MIR.

### Self-reported effect of MIR on mobility and fatigue

We found a statistically significant improvement in the MSWS-12 after MIR in the total cohort (p = 0.002), but also in the subgroups with “mild” and “moderate to severe” walking impairment group (p = 0.014 and p = 0.049, respectively; Table 2).

In line with these results, the majority of pwMS (83%) reported a positive change in overall mobility when completing the GRS at the end of MIR. Twenty percent of pwMS classified the change in mobility as “very much improved”, 30% as “much improved” and 33% as improved. Only 12% of pwMS scored “no change” and 3% “deteriorated”.

MIR was associated with a significant decrease in levels of fatigue in the total cohort (p<0.001). The effect of MIR on fatigue was more pronounced in the subgroup of “mild” (mean change -0.8± 1.4; p = 0.001) as compared to the subgroup with “moderate to severe” walking impairment (mean change -0.5±1.0; p = 0.032) resulting in a strong trend towards a statistical significant difference between the subgroups at the end of MIR (p = 0.051).

## Discussion

The present study explored the ecological validity of improvements in short and long clinical gait tests following MIR in a cohort of 86 pwMS using accelerometry. Due to drop outs 76 pwMS were finally analyzed. While pwMS were able to significantly increase their walking performance when assessed with short and long clinical gait tests, the transferability of these findings to real-life conditions was dependent on pre-existing walking disability determined by EDSS.

In an MS cohort with mild to severe walking disability we were able to document significant walking improvements after four weeks of MIR in walking tests. A recent multicenter study evaluating different mobility outcome measures after rehabilitation reported reference values for clinically meaningful changes of 9.6 and 21.6 meters for the 2MWT and the 6MWT respectively [15]. Mean change levels of 14.8 meters in the 2MWT and 48.9 meters in the 6MWT detected in our cohort clearly exceeded these values and are therefore indicative for having also a daily life impact apart from statistical difference. PwMS also perceived a significant gain in walking ability after MIR as evidenced in the MSWS-12. An improvement of 9.5 is above the recently reported clinically meaningful change after medical treatment [12] and close to changes found after rehabilitation interventions in MS [15], also suggesting a relevant impact on everyday life in our cohort. However, change levels in the T25FW of 0.2 m/s representing a 5.6% improvement were below the commonly accepted threshold for clinically meaningfulness of 20% [10–12], a finding that has also recently been observed in a multicenter rehabilitation study [15].

While significant increases in short and long clinical gait tests after MIR are important results, the primary goal of motor rehabilitation in pwMS is to increase everyday life mobility of pwMS, possibly also leading to improved participation and HRQoL. Using accelerometry as an objective and reliable outcome measure of real-life mobility in the present cohort [22–27], the number of steps performed per day as a marker of real life mobility was found to be comparable to previous studies with pwMS with “mild” walking disability averaging significantly more steps than those with “moderate and severe” walking disability [17,18]. We could demonstrate that an intervention of twenty-eight days of MIR resulted indeed not only in a significant better performance in walking capacity tests, but also in an overall increase in everyday life mobility, a finding that has not been reported so far. Noteworthy, this improvement was strongly correlated to the degree of pre-existing walking disability determined by EDSS. Dichotomising pwMS according to their walking restriction revealed that only pwMS with “mild” walking disability (EDSS<4) were able to transfer improvement in short and long gait tests into their everyday life by increasing also the number of daily steps performed. A multivariate analysis revealed a lower EDSS score, but not age, gender or disease duration to be predictive of a higher gain in everyday mobility after MIR. Therefore, the presence of “moderate to severe” walking impairment seems to be the major limiting factor for translating mobility improvements measured by short and long clinical gait tests after MIR into everyday life in our cohort. Sustained benefit of MIR on everyday mobility as a function of pre-existing walking disability further underscores the notion, that there is not only a window of opportunity for DMTs, but also for early motor rehabilitation in MS [37].

Why do improvements in short and long gait tests are not properly reflected in daily step count as a surrogate for everyday-life mobility in pwMS with “moderate and severe” walking disability? First, the presence of MS-related fatigue is likely to have only a minor effect on short and long walking tests, but can significantly influence everyday-life mobility especially in pwMS with advanced walking disability [17]. Indeed, fatigue levels were lower in the group with “mild” as compared to the group with “moderate and severe walking” disability after MIR, thereby pointing at the potential impact of MS-related fatigue on real-life mobility, that is not depicted in walking capacity tests also in our cohort. Second, the laboratory setting of a standard clinical measurement that is restricted to a quiet environment and flat surfaces [33] is likely to affect its generalizability to real life conditions, especially in pwMS with more severe walking disability [18]. Since everyday life mobility is often challenged, e.g. with distracting noise, barriers, ascents and descents that are much more difficult to overcome for pwMS with walking restrictions, these barriers may limit an improvement in everyday life mobility despite a better performance in walking capacity tests. However, in order to increase everyday life mobility also in pwMS with “moderate and severe” walking disability, these potentially limiting factors have to be thoroughly identified and eliminated wherever possible.

Nevertheless in light of the significant better performance in walking capacity tests, pwMS could have probably also benefitted from an additional behavioral intervention specifically aimed at increasing everyday life mobility as recently has been reported [38,39]. In this context, accelerometry can serve not only as an outcome measure but also as a direct feedback tool for pwMS to enhance mobility.

We used accelerometry as a reference point to investigate the ecological validity of mobility assessments but are aware that this approach has limitations. Wearing an accelerometer itself could potentially alter the behavior of pwMS and might thereby influence the outcome of the results and jeopardize the objectivity of the measurement. However, this restriction seems to be widely negligible when the minimum wearing time exceeds 600 minutes on at least three days a week [34]. Further, the responsiveness of daily steps as a mobility outcome measured by accelerometry may also have influenced the detection of a significant mobility change following MIR in our cohort of pwMS with more pronounced walking disabilities. Ecological validity of short and long walking tests was evidenced to be a function of pre-existing walking disability in the investigated MS cohort. When grouping pwMS according to walking disability we have to acknowledge the smaller sample size of the subgroup with “moderate to severe” walking disability, which limits the generalizability of the findings on the group level at this stage. In addition, although daily step count has been established as walking outcome it cannot precisely differentiate between walking capacity and acitivity behaviour [40]. More sophisticated accelerometer output date like maximum or habitual walking step rate have been reported to better capture real-life walking capacity [41]. The acknowledgment of these new parameters and the implementation of recently published recommendations for the use of mobile technologies in clinical research [42] will potentially result in a clearer picture of real-life walking capacity of pwMS with different degrees of mobility impairment.

In conclusion, this is the first study that longitudinally investigated the ecological validity of short and long clinical gait tests after MIR in pwMS. The impact of significant improvements in the T25FW, the 2MWT and 6MWT on everyday life mobility is dependent on pre-existing walking disability and was restricted to pwMS with “mild” walking disability only.

## Supporting information

S1 Table Comparison of baseline characteristics between included and excluded patients. Data is presented as ^1^mean and standard deviation; ^2^absolute number and percentage; ^3^median and interquartile range. Data is analysed using *independent t-test, **chi-square test or ***Mann-Whitney U test depending on normal distribution. Abbreviations: EDSS, expanded disability status scale.
(DOCX)

S2 Table Quantity of multidisciplinary inpatient rehabilitation (MIR). Data is presented as mean and standard deviation. Differences between walking disability subgroups were evaluated using *chi-square test or **Mann-Whitney U test depending on normal distribution.
(DOCX)
