# Supplementary material for: Ecological validity of walking capacity tests following rehabilitation in people with multiple sclerosis
Source: PLoS One. 2019 Aug 1;14(8):e0220613. doi: 10.1371/journal.pone.0220613 (PMC6675072; doi:10.1371/journal.pone.0220613)
Supplement: S1 Table — Data is presented as 1mean and standard deviation; 2absolute number and percentage; 3median and interquartile range. Data is analysed using *independent t-test, **chi-square test or ***Mann-Whitney U test depending on normal distribution. Abbreviations: EDSS, expanded disability status scale. (DOCX) [file pone.0220613.s001.docx]

**S1 Table. Comparison of baseline characteristics between included and excluded patients.**

|  | included | excluded | *p-value* |
| --- | --- | --- | --- |
| N | 76 | 10 |  |
| Age (years)^1^ | 47.9 (8.3) | 46.8 (11.0) | 0.850* |
| Females^2^ | 49 (64.5) | 5 (50.0) | 0.373** |
| Disease duration (years)^1^ | 11.9 (8.8) | 10.2 (7.8) | 0.657*** |
| Disease subtype^2^ |  |  |  |
| Relapsing remitting | 46 (60.5) | 7 (70.0) | 0.808** |
| Secondary progressive | 13 (17.1) | 1 (10.0) |  |
| Primary progressive | 17 (22.4) | 2 (20.0) |  |
| EDSS^3^ | 3.0 (2.0-5.5) | 3.5 (2.0-5.5) | 0.733*** |
