# Supplementary material for: Ecological validity of walking capacity tests following rehabilitation in people with multiple sclerosis
Source: PLoS One. 2019 Aug 1;14(8):e0220613. doi: 10.1371/journal.pone.0220613 (PMC6675072; doi:10.1371/journal.pone.0220613)
Supplement: S2 Table — Data is presented as mean and standard deviation. Differences between walking disability subgroups were evaluated using *chi-square test or **Mann-Whitney U test depending on normal distribution. (DOCX) [file pone.0220613.s002.docx]

**S2 Table. Quantity of multidisciplinary inpatient rehabilitation (MIR).**

|  |  | Walking disability subgroups | |  |
| --- | --- | --- | --- | --- |
|  | Whole Group | "mild" (EDSS 2-3.5) | "moderate-severe"  (EDSS 4-6.5) | *p*-*value* |
| N | 76 | 49 | 27 |  |
| Individual physiotherapy (min) | 712.2 (222.4) | 685.7 (214.1) | 760.2 (233.2) | 0.164* |
| Group physiotherapy (min) | 845.7 (221.8) | 868.9 (211.1) | 803.7 (238.3) | 0.251** |
| Individual therapy other than physiotherapy (min) | 409.9 (251.9 | 417.3 (247.3) | 396.3 (264.4) | 0.632** |
| Group therapy other than physiotherapy (min) | 310.9 (204.4) | 280.1 (169.1) | 366.7 (250.4) | 0.321** |
